# Malabsorption Syndrome due to Intestinal Amyloidosis as Presentation of Renal Cell Carcinoma

**DOI:** 10.1155/2020/8854620

**Published:** 2020-09-05

**Authors:** Filipe Bessa, Pedro Gaspar, Pedro Antunes Meireles, Maria Inês Parreira, Juliana Serrazina Pedro, Marisa Teixeira da Silva, João Meneses, Rui Victorino, Catarina Mota

**Affiliations:** Clínica Universitária de Medicina II, CHLN-Hospital de Santa Maria, Lisbon, Portugal

## Abstract

Renal cell carcinoma accounts for approximately 3% of adult malignancies. Designated in the literature as “the great masquerader,” the great diversity of clinical manifestations is associated with the several paraneoplastic syndromes that potentially accompany it. Paraneoplastic amyloidosis is described in about 3–8% of cases, only exceptionally as an initial manifestation, with uncommon gastrointestinal involvement. A rare case of malabsorption by intestinal amyloidosis is presented as initial manifestation of renal cell carcinoma, emphasizing the need for early recognition of these paraneoplastic conditions.

## 1. Introduction

Renal cell carcinoma (RCC) originates in the renal cortex, accounting for approximately 3% of adult malignancies and 90–95% of the primary tumors of the kidney [1]. It appears as the 6^th^ most frequent tumor in the Europe, with an incidence of 12.4 new cases per 100,000 inhabitants [2]. The diagnosis is more common between the seventh and eighth decades of life and slightly more frequent in men (M : F, 3 : 2) [3].

The frequent absence of symptoms in the early stages makes the diagnosis usually incidental, with around 25% of patients being asymptomatic [1, 3]. The classic triad of hematuria, flank pain, and palpable abdominal mass is rarely detected, presents only in 10–15% of cases, and is usually a sign of advanced disease [3]. On the contrary, the earlier manifestations may include paraneoplastic syndromes, which arise as a result of the production of several hormones and cytokines by the tumor or adjacent tissues responsible for modulation of the immune system and endocrine dysregulation of the kidney [3]. Among them, the most frequent are hypertension, constitutional symptoms, and hypercalcemia present in 40%, 20–30%, and 10–20% of the cases, respectively [1, 4].

Amyloid A amyloidosis (AA amyloidosis) results from the extracellular deposition of fibrils composed of fragments of the serum apolipoprotein amyloid A, an acute-phase reagent produced by hepatocytes. It appears as a complication of chronic inflammatory diseases such as rheumatoid arthritis and inflammatory bowel disease, chronic infections such as tuberculosis or syphilis, and tumors. As a paraneoplastic syndrome, AA amyloidosis has been described secondarily to renal cell carcinoma, non-Hodgkin's lymphoma, and, less frequently, bladder tumors and gastrointestinal stromal tumors [5, 6].

The clinical manifestations of AA amyloidosis depend on the extent and location of fibers deposition, with amyloid deposits found mostly in the kidney (80% of cases) and, at latter stages of the disease, in the heart and nervous system [7–9]. Gastrointestinal involvement by amyloid deposition in the *muscularis mucosae*, close to the vasculature and nerve plexus, is particularly infrequent [10, 11].

The authors describe a rare case of malabsorption due to intestinal amyloidosis as presentation of renal cell carcinoma.

## 2. Case Presentation

A 60-year-old man, with a previous history of hypertension and smoking habits (30 pack-years), was hospitalized with a four-month history of asthenia and weight loss of 20% of total body weight, associated with diarrhea and epigastric discomfort in the month before admission. No other complaints were registered.

Physical examination revealed marked cachexia (BMI 15.5 kg/m^2^), pallor and dehydration, blood pressure 105/62 mmHg, heart rate 72/min, and respiratory rate 20/min. There were no rashes or skin lesions or palpable lymphadenopathies. Cardiovascular and pulmonary examination was normal. The abdomen was excavated, painful at lower quadrants palpation, and without palpable masses. The limbs exhibited muscle mass atrophy. The remaining examination was unremarkable.

**Figure 1:**
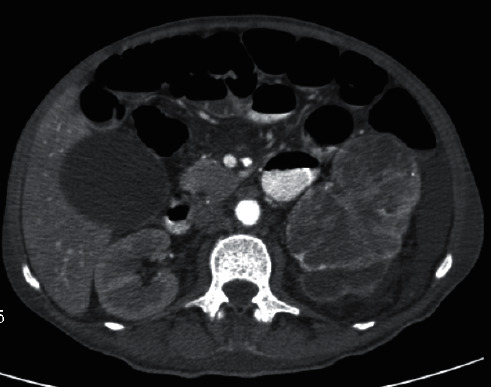
Computed axial tomography of the abdomen with intravenous administration of iodinated contrast: centered on the lower two-thirds of the left kidney, neoformative mass with heterogeneous enhancement, and some areas of liquefaction with greater longitudinal anteroposterior and transversal axis evaluated as 10.5 × 11.7 × 8.5 cm, respectively.

The blood tests revealed normochromic microcytic anemia with a hemoglobin of 12.3 g/dL, leukocytosis (22,5 × 10^9^/L) with neutrophilia (19,8 × 10^9^/L), thrombocytosis (901 × 10^9^/L), elevated C-reactive protein (17.11 mg/dL), renal injury with creatinine of 5.5 mg/dL, urea of 329 mg/dL, hyponatremia of 124 mmol/L, and compensated metabolic acidosis. Renal ultrasound showed a massive solid mass in the left kidney with 12 cm of greater axis. The abdominal computed tomography (CT) revealed on the left kidney, centered to the lower two-thirds, a neoformative mass with heterogeneous enhancement, and some areas of liquefaction, for which the greater dimensions were 10.5 × 11.7 × 8.5 cm longitudinal, anteroposterior, and transversal axis, respectively (Figure 1). It was assumed as the initial hypothesis primary kidney tumor with cachexia/malabsorption syndrome secondary to paraneoplastic syndrome.

**Figure 2:**
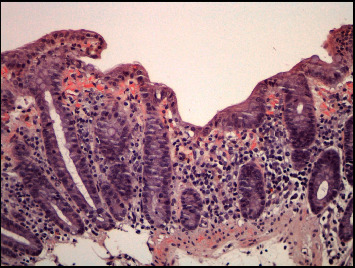
Colon biopsy. Fragment of mucosa from the distal portion of the intestine with conserved general architecture. Deposits of amyloid substance predominantly subepithelial and perivascular, positive for Congo red staining.

**Figure 3:**
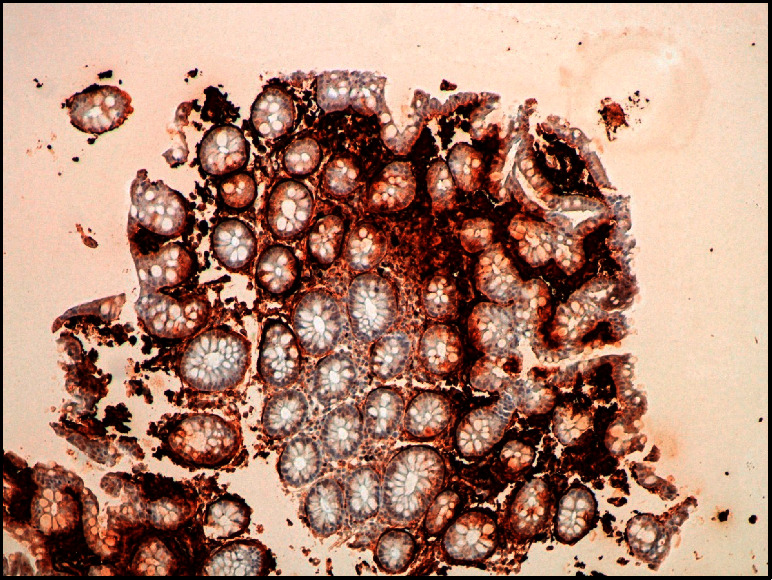
Colon biopsy. Immunohistochemical characterization of amyloid substance deposits, positive for AA amyloid.

During hospitalization, severe anorexia was observed with reduced ingestion, general malaise, and diffuse abdominal discomfort, 8 to 10 diarrheal discharges per day with fatty and bloody content and foamy urine. The remaining investigation revealed hypoalbuminemia of 1.9 g/dl and hypocalcaemia of 7.1 mg/dl, suggesting malabsorption and nephrotic range proteinuria (3 g/24 h). Considering the location and size of the renal tumor and the clinical evolution, with gastrointestinal involvement presenting as malabsorption syndrome and nephrotic-like renal involvement, it was considered the hypothesis of renal cell carcinoma with paraneoplastic amyloidosis. Colonoscopy was performed, showing a diffusely edematous mucosa, some areas with loss of vascular pattern and erythema/spontaneous hemorrhage. The anatomopathological examination of colon biopsies revealed deposits of amyloid substance of predominant subepithelial and perivascular location (Figure 2), and immunohistochemical staining with antibodies specific for the major amyloid precursors (AA, immunoglobulin L chains of *κ* or *λ* type, antitransthyretin) showed positivity only for AA fibrils (Figure 3).

During the investigation, the patient presented with nosocomial pneumonia and was started on empirical antibiotic therapy with piperacillin-tazobactam. The clinical evolution was unfavorable, with refractory septic shock and death on the 8th day of hospitalization.

## 3. Discussion

Typically, RCC remains clinically silent for a long period of the disease course, due in part to the retroperitoneal location of the affected organ [1, 12]. Local symptoms arise only when the tumor is of an adequate size to deflect or invade adjacent structures. The palpable abdominal mass, hematuria, and low back pain triad occurs only in 10–15% of patients, and when present, it is a sign of advanced disease [1]. Notwithstanding, the individual components of this triad are more frequent: hematuria is the most common clinical presentation and is present in 40–60% of cases; flank pain involves distension of the renal capsule and is present in 40% of cases; palpable abdominal mass is found in one-third of patients [1].

Commonly known in the literature as “The Great Masquerader,” RCC is recognized for its frequent association with multisystem paraneoplastic syndromes (10–40% of cases), which require recognition of a wide variety of symptoms [4]. In about one-third of the cases, constitutional symptoms are the first presentation of RCC. Fever occurs in 20–30% of patients, reflecting tumor production of inflammatory cytokines, such as interleukin-6. Tumor production of TNF-*α* alters adipocyte metabolism, clinically resulting in asthenia, cachexia, and weight loss in 66% of patients [13]. Hypercalcemia is one of the most common paraneoplastic syndromes, occurring in about 20% of patients [14]. RCC is still the malignancy most frequently associated with ectopic erythropoietin production (EPO), which occurs in 66% of patients, resulting in erythrocytosis in only 8% of them [1, 15].

AA amyloidosis is a paraneoplastic syndrome rarely associated with RCC (3–8% of cases) although several autopsies have suggested that RCC is the solid malignancy that most often causes AA amyloidosis, accounting for 25–42% of cases of solid tumors with secondary amyloidosis [1, 6, 8, 16]. It is characterized by multisystem extracellular deposition of amyloid fibrils derived from cleavage fragments of the *N*-terminal portion of serum amyloid protein A [7, 17]. The clinical manifestations of AA amyloidosis depend on the extent and location of fibers deposition. Amyloid deposits are found mostly in the kidney (80% of cases), possibly resulting in proteinuria, nephrotic syndrome, and renal injury. Although amyloid deposits are common in the liver and spleen, their clinical relevance is significantly lower in the earlier stages of disease. Cardiac involvement, with restrictive cardiomyopathy associated with heart failure, and neurological involvement occur at a terminal stage of the disease in <2% of cases [7–9]. Involvement of the gastrointestinal tract by AA amyloidosis, such as the case the authors present, is uncommon. Amyloid fibers are deposited in the *muscularis mucosae* of the gastrointestinal tract, near the vasculature and nerve plexus, which increases blood vessel friability, delays peristalsis, and decreases compliance of the intestinal wall, resulting in myopathy and neuromuscular dysfunction [10, 11]. The presence of amyloid in the gastric mucosa mainly determines clinically nausea, vomiting, hematemesis, and epigastric pain. The small intestine is, in the gastrointestinal tract, the organ most affected by amyloid deposits, with associated malabsorption, diarrhea, steatorrhea, hemorrhage, or obstruction. In the colon, amyloid deposition may macroscopically resemble aspects of inflammatory bowel disease, ischemic colitis, or colorectal cancer and may lead to major complications such as stenosis, bleeding, obstruction, and perforation [10, 11, 18].

The first association between amyloidosis and renal cell carcinoma was described by Ask-Up-mark in 1940 [8]. We found in the literature 19 cases of AA amyloidosis secondary to RCC [6]. Amyloid deposits are documented in the kidney, gastrointestinal tract, retroperitoneum, liver, spleen, adrenal glands, conjunctiva, and upper respiratory tract mucosa. Renal involvement was the most common presentation of AA amyloidosis. Of the 19 published cases, 14 had amyloid deposits in the kidney, with associated hematuria, nephrotic syndrome, and acute renal injury [6]. Amyloidosis with gastrointestinal involvement was found in four of the nineteen cases, resulting in diarrhea, abdominal discomfort, and weight loss. As inaugural presentation of RCC, gastrointestinal amyloidosis is reported in only two cases.

It has been shown that RCC-associated paraneoplastic syndromes are not a marker of advanced disease or poor prognosis, usually remitting with tumor removal. Early recognition of the symptoms associated with these conditions is, therefore, of particular importance and may provide early diagnosis and treatment. Paraneoplastic amyloidosis is only exceptionally an initial manifestation, with uncommon intestinal involvement. A rare case of malabsorption due to intestinal paraneoplastic amyloidosis is presented as initial manifestation of renal cell carcinoma.
